# Fused multi-modal similarity network as prior in guiding brain imaging genetic association

**DOI:** 10.3389/fdata.2023.1151893

**Published:** 2023-05-05

**Authors:** Bing He, Linhui Xie, Pradeep Varathan, Kwangsik Nho, Shannon L. Risacher, Andrew J. Saykin, Jingwen Yan, The Alzheimer's Disease Neuroimaging Initiative

**Affiliations:** ^1^Luddy School of Informatics, Computing, and Engineering, Indiana University Purdue University Indianapolis (IUPUI), Indianapolis, IN, United States; ^2^School of Engineering and Technology, Indiana University Purdue University Indianapolis (IUPUI), Indianapolis, IN, United States; ^3^Indiana Alzheimer's Disease Research Center, Indianapolis, IN, United States

**Keywords:** Alzheimer's disease, imaging genetics, network fusion, prior knowledge, DSCCA

## Abstract

**Introduction:**

Brain imaging genetics aims to explore the genetic architecture underlying brain structure and functions. Recent studies showed that the incorporation of prior knowledge, such as subject diagnosis information and brain regional correlation, can help identify significantly stronger imaging genetic associations. However, sometimes such information may be incomplete or even unavailable.

**Methods:**

In this study, we explore a new data-driven prior knowledge that captures the subject-level similarity by fusing multi-modal similarity networks. It was incorporated into the sparse canonical correlation analysis (SCCA) model, which is aimed to identify a small set of brain imaging and genetic markers that explain the similarity matrix supported by both modalities. It was applied to amyloid and tau imaging data of the ADNI cohort, respectively.

**Results:**

Fused similarity matrix across imaging and genetic data was found to improve the association performance better or similarly well as diagnosis information, and therefore would be a potential substitute prior when the diagnosis information is not available (i.e., studies focused on healthy controls).

**Discussion:**

Our result confirmed the value of all types of prior knowledge in improving association identification. In addition, the fused network representing the subject relationship supported by multi-modal data showed consistently the best or equally best performance compared to the diagnosis network and the co-expression network.

## 1. Introduction

Brain imaging genetics studies the influence of genetic variation on brain structure and function. Its major task is to examine the association between genetic markers such as single nucleotide polymorphisms (SNPs) and quantitative traits (QTs) extracted from multi-modal neuroimaging data (e.g., MRI and PET scans). Although both gene and imaging phenotype are two well-known factors contributing to brain function, exploring their underlying connections would lead to a better mechanistic understanding of normal or disordered brain functions.

Early studies in brain imaging genetics associations typically adopt a univariate approach (Shen et al., 2010), where each pair of SNP and brain phenotype were examined individually for the association. Based on the assumption that a real imaging genetic association typically involves a small subset of SNPs and QTs, bi-multivariate association models, such as sparse canonical correlation analysis (SCCA), have been increasingly used later to identify the best linear transformation for imaging and genetics features so that the correlation between imaging and genetic components can be maximized (Chi et al., 2013; Lin et al., 2014). Recently, to further improve the performance, various prior knowledge, such as diagnosis group, linkage disequilibrium block in SNPs, and brain co-expression networks, have been incorporated into the SCCA model. These prior knowledge mitigates the effect of limited sample size and all of them have helped yield much improved performance over the traditional SCCA model. However, these prior knowledge are not always available or sometimes not applicable. For example, the brain co-expression network used by Yan et al. (2014) requires a predefined subset of genes related to brain imaging, which would not be available for structural MRI. Some imaging genetics studies may have to deal with data without diagnosis information or with a single diagnosis group. In both cases, diagnosis information cannot be used as prior. Data-driven subject similarity network has been previously explored but was derived from a single modality with limited guidance (Du et al., 2016).

To address this problem, we propose a multi-modal subject similarity network as a new prior knowledge using the similarity network fusion (SNF) approach. In particular, we aim to build a subject similarity network that is supported by both brain imaging phenotype and genetic variants. Then, we will employ a discriminative SCCA model (Yan et al., 2017) to identify a subset of SNPs and brain imaging ROIs that are not only highly correlated but also can best explain the shared similarity network. When applied to the real brain imaging (including amyloid and tau PET) and genetic data in the ADNI cohort, we found that SCCA guided by the fused similarity network showed similar performance as that guided by diagnosis information and both outperformed those guided by other prior knowledge. Taken together, our results suggested the value of a fused similarity network as a great alternative prior in case of the absence of diagnostic network, particularly when the study focuses only on one group (like the aging process of healthy older adults).

## 2. Data

Amyloid and tau PET imaging data, together with the imputed genotype data, were downloaded from the Alzheimer's disease Neuroimaging Initiative (ADNI) (http://adni.loni.usc.edu/) database. The ADNI was launched in 2003 as a public-private partnership, led by Principal Investigator Michael W. Weiner, MD. The primary goal of ADNI has been to test whether serial magnetic resonance imaging (MRI), positron emission tomography (PET), other biological markers, and clinical and neuropsychological assessments can be combined to measure the progression of mild cognitive impairment (MCI) and early Alzheimer's disease (AD). For up-to-date information, see www.adni-info.org. In this study, we have 800 subjects with both genotype data and amyloid imaging, including 158 cognitive normals (CN), 90 with significant memory concern (SMC), 279 early mild cognitive impairment (EMCI), 143 late MCI (LMCI), and 130 AD patients. For Tau, we have 291 subjects with both genotype data and tau imaging data, including 75 CN, 135 SMC, 30 EMCI, 32 LMCI, and 19 AD patients. The detailed demographic information of gender, age, and education years are shown in Table 1.

**Table 1:** Demographic information of ADNI image data.

	**Subjects**	**NC**	**SMC**	**EMCI**	**LMCI**	**AD**
Amyloid	Number	158	90	279	143	130
	Gender (M/F)	79/79	36/54	158/121	79/64	78/52
	Age (mean ± SD)	73.25 ± 6.05	71.62 ± 5.45	71.05 ± 7.27	71.41 ± 7.49	73.95 ± 8.03
	Educ (mean ± SD)	16.62 ± 2.50	16.79 ± 2.62	16.09 ± 2.66	16.71 ± 2.51	15.72 ± 2.69
TAU	Number	75	135	30	32	19
	Gender (M/F)	27/48	55/80	18/12	20/12	12/7
	Age (mean ± SD)	69.25 ± 5.40	70.96 ± 6.15	70.2 ± 7.23	71.97 ± 8.64	73.42 ± 10.80
	Edu (mean ± SD)	17.12 ± 2.14	16.84 ± 2.21	16.03 ± 2.68	15.94 ± 2.23	16.16 ± 2.75

### 2.1. Imaging data preprocessing

Both amyloid and tau imaging data have been downloaded from the ADNI website as preprocessed. Briefly, amyloid PET used florbetapir (18F) as a tracer to measure amyloid-β (Aβ) plaques (Okamura and Yanai, 2010). For each subject, brain regions of interest (ROIs) were defined from structural MRI through segmentation and parcellation using Freesurfer (Fischl, 2012). Then, each florbetapir scan was coregistered to the corresponding MRI and calculated the mean florbetapir uptake within the predefined ROIs. All the regional amyloid deposition was re-normalized using the whole cerebellum as a reference region. Tau PET used flortaucipir as a tracer to detect the aggregated tau (Fleisher et al., 2020), and the regional tau aggregation was obtained similarly as amyloid. All the regional tau tangle accumulation was re-normalized using inferior cerebellar as reference region. Finally, we have amyloid measurement in 68 cortical ROIs and tau measurement in 73 ROIs. More detailed image processing information can be found in Landau et al. (2013) and Landau et al. (2016). To remove potential bias, both amyloid and tau measures were pre-adjusted using baseline age, gender, and the weight derived from healthy controls. Finally, they were normalized to zero mean and unit variance for subsequent analysis.

### 2.2. Genotype data processing

Genotype data of both ADNI-1 and ADNI-2/GO phases were also obtained from the ADNI cohort (adni.loni.usc.edu). We focused our analysis on top SNPs from the International Genomics of Alzheimer's Project (IGAP), a large-scale genome-wide association study of AD (Schellenberg and IGAP, 2012). It tested the association of 7,055,881 single nucleotide polymorphisms (SNPs) of 17,008 Alzheimer's disease cases and 37,154 controls. SNPs with *p* ≤ 5 × 10^−6^ in their meta analysis were used as our candidates and their genotypes were extracted based on the quality controlled and imputed genetic data in the ADNI using PLINK (Purcell et al., 2007). Finally, we have 1,080 SNPs for the subsequent imaging genetics association.

## 3. Methods

To evaluate the proposed prior knowledge, we apply it to amyloid and tau imaging and genetic data in the study of Alzheimer's disease (AD). Deposition of amyloid-β and abnormal accumulations of tau protein are two major hallmarks in AD pathogenesis. Prior knowledge tested in this analysis for comparison include (1) subject diagnosis information, (2) brain co-expression network using amyloid- and tau-related genes, respectively (Zeng et al., 2012), and (3) fused similarity network built on imaging and genetics data.

### 3.1. Fused similarity network

**Figure 1:**
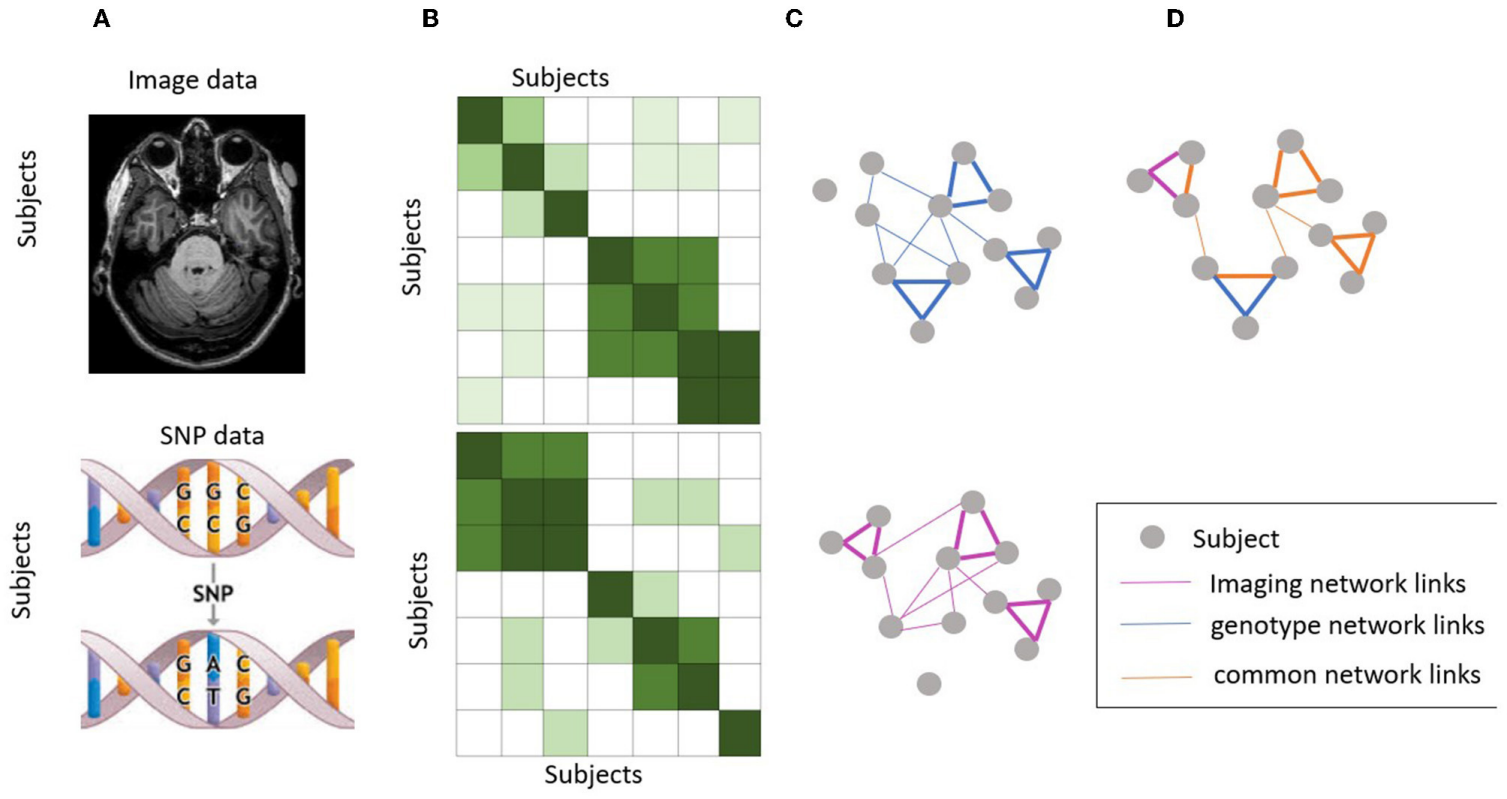
Workflow of similarity network fusion. **(A)** Original SNP data and imaging data. **(B)** Subject similarity matrix generated using normalized mutual information (NMI). **(C)** Subject similarity network (equivalent to the subject by subject matrix). **(D)** Fused network by integrating information from both imaging and genotype data.

In this study, we proposed to use a fused similarity network as a new prior knowledge, as inspired by Wang et al. (2014) and hypothesize that it will help improve the performance of imaging genetics association. First, we have original SNP data and imaging data showed in Figure 1A, we build a sample-sample similarity matrix for imaging and genetic data, respectively (Figure 1B) and their subject similarity network look like in Figure 1C. This similarity matrix can be seen as a similarity network G = (V,E,W), where nodes V represent subjects {***x***_1_, ***x***_2_, ..., ***x***_*n*_}, the weighted edges *E* represent similarities of a subject to others and W is a *n* × *n* similarity weighted matrix representing the similarity between subjects ***x***_*i*_ and ***x***_*j*_. Suppose ρ (***x***_*i*_,***x***_*j*_) is euclidean distance between subjects ***x***_*i*_ and ***x***_*j*_. Then a scaled exponential similarity kernel was used to determine the weight of the edge:

(1) $$\begin{array}{l} \text{W}\left(i,j\right)=\exp\left(-\frac{\rho^{2}\left(x_{i},x_{j}\right)}{\mu \varepsilon_{i,j}}\right) \end{array}$$

where μ is a hyper parameter that can be empirically set. It was recommended from [0.3, 0.8], and we set it as 0.5 by default (Wang et al., 2014). and ε_*i, j*_ is used to eliminate the scaling problem. Here we define:

(2) $$\begin{array}{l} \varepsilon_{i,j}=\frac{\mathrm{mean}\left(\rho \left(x_{i},N_{i}\right)\right)+\mathrm{mean}\left(\rho \left(x_{j},N_{j}\right)\right)+\rho \left(x_{i},x_{j}\right)}{3} \end{array}$$

where *N*_*i*_ denote a set of *x*_*i*_'s neighbors including *x*_*i*_ in *G*, and ρ(*x*_*i*_, *N*_*i*_) is the average value of the distance between *x*_*i*_ and each of its neighbors. Each row of **W** was then normalized as below:

(3) $$\begin{array}{l} \text{P}\left(i,j\right)=\{\begin{array}{c} \frac{\text{W}\left(i,j\right)}{2\Sigma_{k\neq i}\text{W}\left(i,k\right)},j\neq i\\ 1/2,j=i \end{array} \end{array}$$

Given a graph *G*, we use *K* nearest neighbors (KNN) to measure local affinity as:

(4) $$\begin{array}{l} \text{S}\left(i,j\right)=\{\begin{array}{cc} \frac{\text{W}\left(i,j\right)}{\Sigma_{k\in N_{i}}\text{W}\left(i,k\right)}, & j\in N_{i}\\ 0 & \text{otherwise} \end{array} \end{array}$$

**P** offers the similarity information of each subject to all others and **S** encodes the similarity to the **K** most similar neighbors for each subject. In this article, we have two types of data, genomics data and imaging data. We first calculated the status matrices *P*^(1)^ and *P*^(2)^ following equation (3), and then the kernel matrices *S*^(1)^ and *S*^(2)^ following in equation (4). For both amyloid and tau data, we tested the model performance with varying *K* values from 5 to 50. Association performance was found highly stable across varying *K* values, therefore we set *K* = 20 as default.

Next, we performed the network fusion of two kernel matrices using a message-passing theory (Pearl, 1988) non-linear method. This is an iterative process where both networks keep getting updated until they converge (i.e., not change much). The final network, known as the fused network, is expected to represent the subject relationships supported by both brain image data and genotype data. Let $P_{t=0}^{\left(1\right)}=P^{\left(1\right)}$ and $P_{t=0}^{\left(2\right)}=P^{\left(2\right)}$ be the initial two status matrices when *t* = 0. The fusion process will iteratively update two similarity matrices corresponding to two data types as follows:

(5) $$\begin{array}{l} {\text{P}}_{t+1}^{\left(1\right)}={\text{S}}^{\left(1\right)}\times {\text{P}}_{t}^{\left(2\right)}\times {\left({\text{S}}^{\left(1\right)}\right)}^{T} \end{array}$$

(6) $$\begin{array}{l} {\text{P}}_{t+1}^{\left(2\right)}={\text{S}}^{\left(2\right)}\times {\text{P}}_{t}^{\left(1\right)}\times {\left({\text{S}}^{\left(2\right)}\right)}^{T} \end{array}$$

where ${\text{P}}_{t+1}^{\left(1\right)}$ and ${\text{P}}_{t+1}^{\left(2\right)}$ are the status matrix of these two data types after t iterations. After each iteration, we performed normalization on ${\text{P}}_{t+1}^{\left(1\right)}$ and ${\text{P}}_{t+1}^{\left(2\right)}$ as in equation (3). This step ensures that subject self-similarity is always higher than the similarity to other neighbors. Here, the alternating multiplication of the squared KNN similarity of the two modalities essentially combines the local information of the two modalities in a way that reinforces their shared information. By multiplying the initial similarity matrix of one modality with the squared KNN similarity matrix of the other modality, the shared information between the two modalities is amplified and the unique information in each modality is retained. This process is then repeated in an alternating manner to ensure that both modalities contribute equally to the final similarity matrix, thereby achieving a balanced fusion of the two modalities (The fused network as showed in Figure 1D). This approach is expected to result in a more informative similarity matrix that captures the shared and unique features of both modalities, which in turn can improve the performance of downstream analysis such as association identification between brain imaging and genetic features.

### 3.2. Prior knowledge for comparison

#### 3.2.1. Diagnosis network

A similarity matrix based on diagnosis was built by assigning 1 s between samples in the same diagnosis and 0 s otherwise. In other words, we build a complete graph for all the subjects belonging to the same diagnosis group. To ensure Σ_*j*_**P**(*i, j*) = 1, it was then normalized by setting the diagonal entries as 0.5, and other elements as 0.5 divided by the group size.

(7) $$\begin{array}{l} \text{P}\left(i,j\right)=\{\begin{array}{c} \frac{1}{2\cdot Length\left(Group\left(k\right)\right)},j\neq i\\ 1/2,j=i \end{array} \end{array}$$

where *Length*(*Group*(*k*)) means the size of diagnosis group, and there are totally five groups in this article.

#### 3.2.2. Brain co-expression network

We use amyloid as an example to demonstrate the co-expression network construction process. We first identified 15 genes related to amyloid pathways according to previous studies (Swaminathan et al., 2012). We then extracted the expression level of these genes across 1,210 brain samples in the Allen Human Brain Atlas (AHBA) database. A partial correlation analysis was performed on the brain expression data, and generated a 1, 210 × 1,210 matrix indicating the ROI–ROI similarity based on the expression of selected genes. This matrix was later down-sampled to 68 × 68, where all 1,210 brain samples were mapped to amyloid ROIs and the median value was applied to aggregate the similarity measures. For tau, there are eight genes found involved in tau phosphorylation pathway (Bekris et al., 2012). We went through the same process and generated a 73 × 73 co-expression matrix for tau. These two matrices were used as the prior knowledge in subsequent analysis.

### 3.3. Discriminative SCCA

In this part, let $X=\left\{x_{1},x_{2},...,x_{n}\right\}\subseteq R^{p}$ be the imaging data and $Y=\left\{y_{1},y_{2},...,y_{n}\right\}\subseteq R^{q}$ be the genotype data, where n is the number of patients, *p* and *q* are the numbers of ROIs and SNPs, respectively. Sparse canonical correlation analysis (SCCA) aims to find the maximal correlation between *Xu* and *Yv* by adjusting these two weights, u and v, which indicates the significance of each feature of the imaging genetic associations. As shown in this formula:

(8) $$\begin{array}{l} \begin{array}{c} \underset{\text{u},\text{v}}{\max}{\text{u}}^{T}{\text{X}}^{T}\text{Y}\text{v}\\ \;\text{s}.t.{\text{u}}^{T}{\text{X}}^{T}\text{X}\text{u}=1,{\text{v}}^{T}{\text{Y}}^{T}\text{Y}\text{v}=1,P_{1}\left(u\right)\leq c1,P_{2}\left(v\right)\leq c2 \end{array} \end{array}$$

where ***P***_1_(*u*) ≤ *c*1*and**P***_2_(*v*) ≤ *c*2 are two penalty terms to control the sparsity of selected features. In this study, we used the PMA package (Witten et al., 2009) that applied the *L*_1_ norm penalty for *P*_1_ and *P*_2_ constraints to perform the SCCA method. To ensure the selection of disease-relevant features, we used a novel discriminative SCCA (DSCCA) algorithm (Yan et al., 2017) to integrate imaging data, SNPs data and the prior knowledge for imaging genetics association. Prior knowledge can be diagnosis network, fusion network or ROI–ROI co-expression network. As such, we can not only identify disease-relevant multi-modal biomarkers, but also reveal a strong association between them. Finally, we compare the performance of multiple DSCCA models guided by different prior knowledge.

For the original DSCCA algorithm, there are two constraints, *P*_1_ and *P*_2_, which are added for the multi-class discrimination, inspired by the application of locality preserving projection (LPP) in linear discriminative analysis (Ghamisi et al., 2018).

(9) $$\begin{array}{l} \begin{array}{c} P_{1}\left(\text{u}\right)=||\text{u}|{|}_{D}={\text{u}}^{T}{\text{X}}^{T}{\text{L}}_{w}\text{X}\text{u}\\ P_{2}\left(\text{v}\right)=||\text{v}|{|}_{D}={\text{v}}^{T}{\text{Y}}^{T}{\text{L}}_{w}\text{Y}\text{v} \end{array} \end{array}$$

Here, *L*_*w*_ is the Laplacian graphs of prior knowledge graph.

The final objective function of DSCCA can be written as follows:

(10) $$\begin{array}{l} \begin{array}{c} \underset{\text{u},\text{v}}{\max}{\text{u}}^{T}{\text{X}}^{T}\text{Y}\text{v}-\frac{\beta_{1}}{2}P_{1}\left(\text{u}\right)-\frac{\beta_{2}}{2}P_{2}\left(\text{v}\right)\\ s.t.{\text{u}}^{T}{\text{X}}^{T}\text{X}\text{u}=1,{\text{v}}^{T}{\text{Y}}^{T}\text{Y}\text{v}=1,||\text{u}|{|}_{1}\leq c_{1},||\text{v}|{|}_{1}\leq c_{2} \end{array} \end{array}$$

Using Lagrange multipliers, Equation (10) can be reformulated as follows:

(11) $$\begin{array}{l} \underset{\text{u},\text{v}}{\max}{\text{u}}^{T}{\text{X}}^{T}\text{Y}\text{v}-\frac{\gamma_{1}}{2}||\text{X}\text{u}|{|}_{2}^{2}-\frac{\gamma_{2}}{2}||\text{Y}\text{v}|{|}_{2}^{2}-\frac{\beta_{1}}{2}P_{1}\left(\text{u}\right)-\frac{\beta_{2}}{2}P_{2}\left(\text{v}\right)\\ -\lambda_{1}||\text{u}|{|}_{1}-\lambda_{2}||\text{v}|{|}_{1} \end{array}$$

Equation (11) is known as a bi-convex problem, which can be solved using an alternating algorithm as discussed in Witten et al. (2009). By fixing *u* and *v*, respectively, we will have the following two minimization problems shown in Equations (12) and (13).

(12) $$\begin{array}{l} \underset{\text{u}}{\min}-{\text{u}}^{T}{\text{X}}^{T}\text{Y}\text{v}+\frac{\gamma_{1}}{2}{\text{u}}^{T}{\text{X}}^{T}\text{X}\text{u}+\frac{\beta_{1}}{2}P_{1}\left(\text{u}\right)+\lambda_{1}||\text{u}|{|}_{1} \end{array}$$

(13) $$\begin{array}{l} \underset{\text{v}}{\min}-{\text{u}}^{T}{\text{X}}^{T}\text{Y}\text{v}+\frac{\gamma_{2}}{2}{\text{v}}^{T}{\text{Y}}^{T}\text{Y}\text{v}+\frac{\beta_{2}}{2}P_{2}\left(\text{v}\right)+\lambda_{2}||\text{v}|{|}_{1} \end{array}$$

We used the Nesterovs accelerated proximal gradient optimization algorithm to solve this objective function following original DSCCA paper (Liu et al., 2012; Yan et al., 2017). The convergence is based on the value changes of the objective function and we use 10^−6^ as stop criteria. A five-fold nested cross-validation was applied to automatically tune the parameters ***β***_1_, ***β***_2_, ***λ***_1_, and ***λ***_2_. According to Chen et al. (2012), the learned pattern and performance are insensitive to ***γ***_1_ and ***γ***_2_ settings. Therefore, in this article, we set both of them to 1 for simplicity.

## 4. Results

To test the effect of different prior knowledge on the performance of imaging genetics association, we performed four groups of experiments including the DSCCA algorithm with different prior knowledge (fusion network, diagnosis network, and ROI–ROI network) and the simple SCCA method as the baseline. For SCCA, the parameters were automatically tuned using a permutation method provided in the PMA package. For DSCCA algorithm, we applied a five-fold nested cross-validation to tune the parameters that can also help avoid the overfitting problem. For a fair comparison, the training/test partition was kept exactly the same across methods and ratios of diagnosis groups inside each partition are also identical. All methods went through the same nested cross-validation for parameter tuning.

### 4.1. Imaging genetic associations for AV45

We first tested the association between brain-wide amyloid deposition and top AD-risk SNPs. The performance of test data including the DSCCA algorithm with fused network, diagnosis network, co-expression network as prior knowledge, and the original SCCA method are shown in Table 2. As expected, DSCCA algorithms with the guidance of prior knowledge all outperformed traditional SCCA, confirming that prior knowledge does help reveal stronger brain imaging genetics associations. More specifically, out of all three types of prior, fused network and diagnosis network led to similar association performance, which is much better than the co-expression network.

**Table 2:** Test performance of brain imaging genetics association on AV45.

**Prior knowledge**	**Testing results**					
	**Fold 1**	**Fold 2**	**Fold 3**	**Fold 4**	**Fold 5**	**Average**
**Fused network**	**0.4872**	**0.5844**	**0.5959**	**0.5586**	**0.5043**	**0.5461**
Diagnosis network	0.4863	0.5848	0.5919	0.5655	0.4945	0.5446
Co-expression network	0.3486	0.4401	0.4185	0.4203	0.37	0.3995
SCCA (no prior)	0.2838	0.4041	0.4015	0.3588	0.3865	0.367

Bold values denote the best performance.

**Figure 2:**
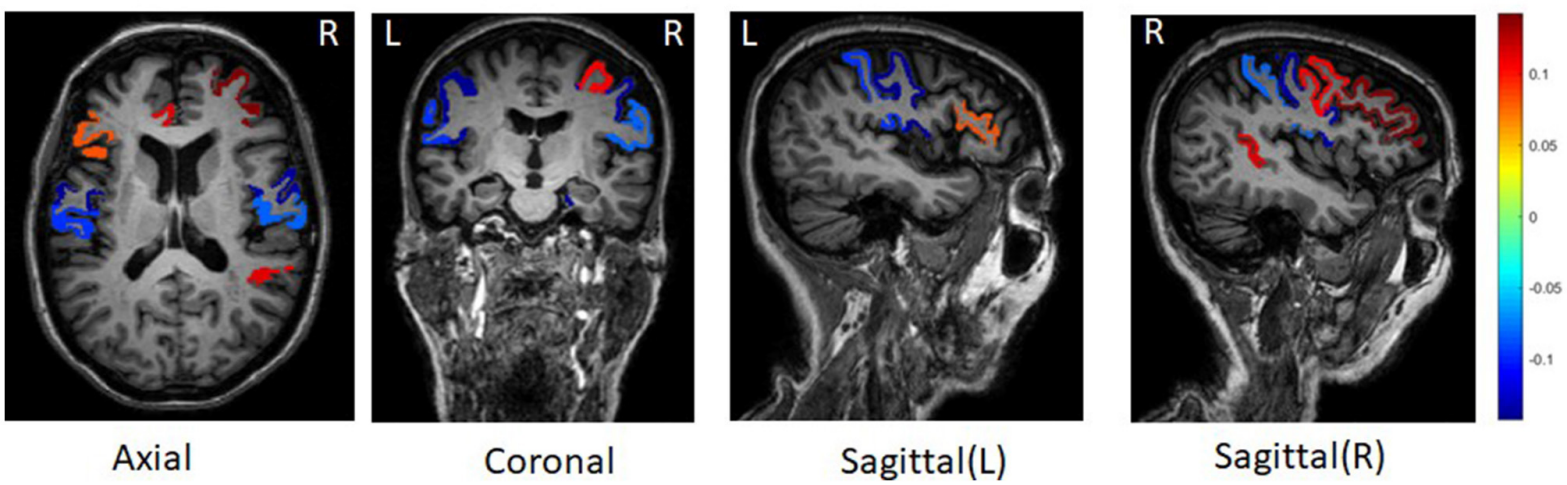
Top 10 brain regions with amyloid deposition associated with *APOE SNP rs429358*.

For all ROIs and SNPs, we averaged their weights across five-folds for feature selection. Figure 2 shows the top 10 ROIs selected by DSCCA guided by the fused network, including left precentral, right parahippocampal, right rostral middle frontal, right precentral, right bankssts, left rostral anterior gingulate, right caudal middle frontal, left postcentral, right postcentral, and left parstriangularis. Among these, right bankssts, right caudal middle frontal, parstriangularis, and left rostral anterior gingulate are part of the default mode network (DMN), and right rostral middle frontal is part of the frontoparietal network. Both of them have consistently shown early accumulation of cortical Aβ fibrils in previous studies (Palmqvist et al., 2017). Amyloid deposition of these regions is strongly associated with only one SNP, *APOE SNP (rs429358)*, which is known as the major risk factor for Alzheimer's disease. There are strong evidences suggesting that *APOE* could inhibit amyloid-β (Aβ) clearance and promote Aβ aggregation to increase AD risk (Polvikoski et al., 1995; Kim et al., 2009; Kok et al., 2009; Wirths, 2010).

### 4.2. Imaging genetic associations for tau

We next tested the association between brain-wide tau deposition and top AD risk SNPs. The performance on test data across all the methods is shown in Table 3. Similar to amyloid, DSCCA guided by three types of prior knowledge performed significantly better than SCCA. Also, all tree DSCCA models showed very similar performance. Considering that the co-expression network only showed moderate performance in amyloid data, we speculate that the selection of candidate genes has a major effect on the prior co-expression network and later lead to the fluctuation of association performance.

**Table 3:** Tau 5-fold cross-validation results.

**Prior knowledge**	**Testing results**					
	**Fold 1**	**Fold 2**	**Fold 3**	**Fold 4**	**Fold 5**	**Average**
**Fused network**	**0.4186**	**0.3948**	**0.4882**	**0.1725**	**0.5443**	**0.4025**
Diagnosis network	0.4237	0.3786	0.4809	0.1722	0.5458	0.4002
Co-expression network	0.4251	0.4246	0.4785	0.1775	0.5424	0.4096
SCCA (no prior)	0.3084	0.2674	0.321	0.1491	0.581	0.3254

Bold values denote the best performance.

**Figure 3:**
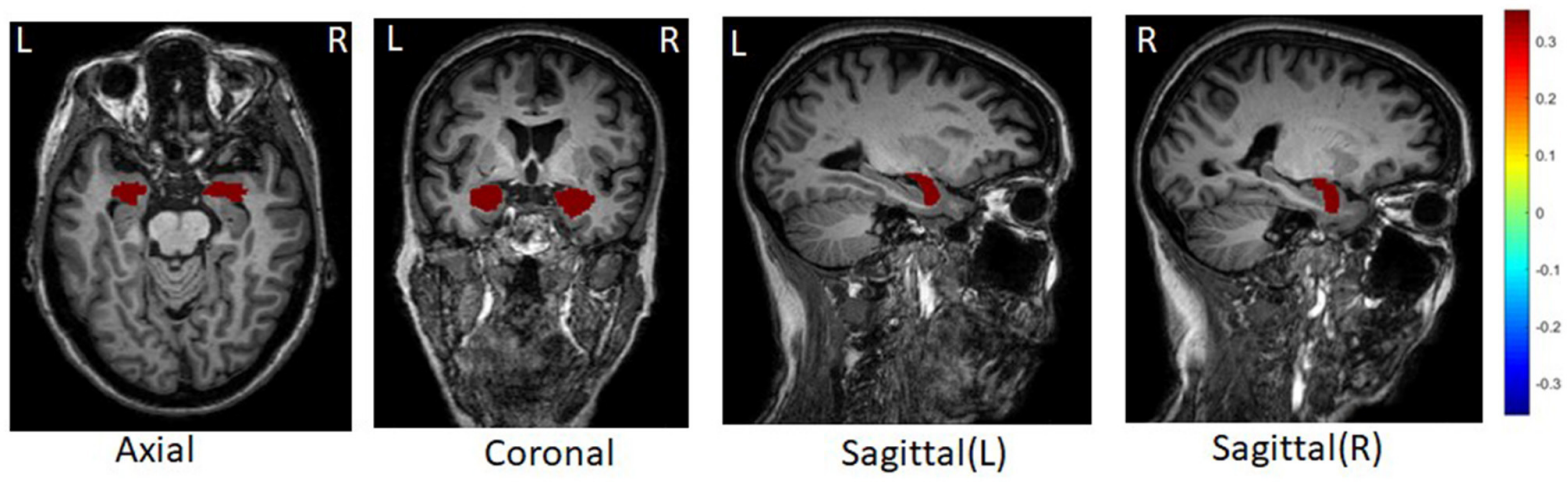
Top tau brain regions selected by DSCCA guided by fused network.

After averaging the weight across 5-folds, we found the tau deposition in the left and right amygdala are strongly associated with 56 SNPs (Figure 3). Amygdala is one of the earliest sites showing tau deposition and neurofibrillary tangles, as reported in previous post-mortem and neuroimaging studies (Vogt et al., 1990; Abiose et al., 2020; Insel et al., 2020). Early tau position in Amygdala is associated with reduced volume and worse cognition performance as well in the preclinical stage of AD (Abiose et al., 2020; Berron et al., 2021). Selected 56 SNPs associating with the amygdala tau deposition are located in or near genes *TOMM40, AC011481.3, NECTIN2, APOC1, AC011481.2, AC015687.1, CLU*. *TOMM40* poly-T lengths have a significant relationship with the higher medial temporal plaque and tangle burden in the living brain of non-demented older adults within individuals not carrying the *APOE*-4 allele (Siddarth et al., 2018). We further performed pathway enrichment analysis of these genes using EnrichR (Kuleshov et al., 2016). Top enriched biological process in gene ontology include regulation of receptor-mediated endocytosis (adjusted *p* = 0.01) and cholesterol transport (adjusted *p* = 0.01).

## 5. Discussion

We proposed a new data-driven prior knowledge and tested whether it could help improve the performance of association between brain imaging and genetic features. Our result confirmed the value of all types of prior knowledge in improving association identification. In addition, the fused network representing the subject relationship supported by multi-modal data showed consistently the best or equally best performance compared to the diagnosis network and the co-expression network. By incorporating information from multiple modalities, the fused network more accurately captures the similarity of subjects in disease severity. With the guidance of such a network, it is more likely to reveal the imaging genetic associations related to AD. Co-expression network showed moderate performance in amyloid but was among the top performers in Tau, suggesting the potential effect of gene selection. The construction of the co-expression network relies on a careful selection of gene set that is specific to certain aspects of AD. While our knowledge of the biological mechanism underlying AD is still very limited, there is no optimal strategy to select relevant genes and different selection processes may lead to varying performance in association. Therefore, the value of co-expression network as prior is compromised. Finally, considering fused network and diagnosis network consistently demonstrated similarly good performance, fused network can be valuable prior knowledge to leverage when there is no diagnosis information or in case of studies using a single diagnosis group, e.g., control only or case only.

## Data availability statement

The original contributions presented in the study are included in the article/supplementary material, further inquiries can be directed to the corresponding author.

## Author contributions

BH: conceptualization, methodology, visualization, formal analysis, validation, writing—original draft, and writing—review and editing. LX: investigation, visualization, and formal analysis. PV: conceptualization and visualization. KN: data curation and writing—review and editing. SR: methodology, supervision, and writing—review and editing. AS: data curation, resource, and writing—review and editing. JY: conceptualization, methodology, visualization, writing—original draft, writing—review and editing, supervision, and funding acquisition. All authors contributed to the article and approved the submitted version.
